# Charging of Piezoelectric Cellular Polypropylene Film by Means of a Series Dielectric Layer

**DOI:** 10.3390/polym13030333

**Published:** 2021-01-21

**Authors:** Pedro Llovera-Segovia, Gustavo Ortega-Braña, Vicente Fuster-Roig, Alfredo Quijano-López

**Affiliations:** 1 Instituto de Tecnología Eléctrica, Universitat Politècnica de València, Camino de Vera s/n, 46022 Valencia, Spain; vfuster@ite.upv.es (V.F.-R.); alfredo.quijano@ite.es (A.Q.-L.); 2 Instituto Tecnológico de la Energía (ITE), Carrer de Juan de la Cierva y Codorniu 24, Paterna, 46980 Valencia, Spain; 3 Independent Researcher, Calle Lérida 31, 46009 Valencia, Spain; gustavortegab@gmail.com

**Keywords:** piezoelectric polymers, polymer foams, electrets

## Abstract

Piezoelectric polymer cellular films have been developed and improved in the past decades. These piezoelectric materials are based on the polarization of the internal cells by means of induced discharges in the gas inside the cells. Internal discharges are driven by an external applied electric field. With this polarization method, cellular polypropylene (PP) polymers exhibit a high piezoelectric coefficient d_33_ and have been investigated because of their low dielectric polarization, high resistivity, and flexibility. Charging polymers foams is normally obtained by applying a corona discharge to the surface with a single tip electrode-plane arrangement or a triode electrode, which consists of a tip electrode-plane structure with a controlled potential intermediate mesh. Corona charging allows the surface potential of the sample to rise without breakdown or surface flashover. A charging method has been developed without corona discharge, and this has provided good results. In our work, a method has been developed to polarize polypropylene foams by applying an insulated high-voltage electrode on the surface of the sample. The dielectric layer in series with the sample allows for a high internal electric field to be reached in the sample but avoids dielectric breakdown of the sample. The distribution of the electric field between the sample and the dielectric barrier has been calculated. Experimental results with three different electrodes present good outcome in agreement with the calculations. High d_33_ constants of about 880 pC/N have been obtained. Mapping of the d_33_ constant on the surface has also been carried out showing good homogeneity on the area under the electrode.

## 1. Introduction

Piezoelectric cellular polymer films have been studied since 1989 [1]. These polymers exhibit piezoelectric behavior after a pooling treatment [2]. By means of an external applied field, internal electrical discharges are driven in the cells. As a consequence, a static charge appears in the walls of the voids, creating internal dipoles [3,4]. A dielectric barrier discharge (DBD) model has been used to explain the mechanism of internal voids charging in polymer foams [5]. DBD has been used in several applications during recent decades [6]. This kind of discharges is a plasma generated between two electrodes with a dielectric in the middle. Dielectric material between both electrodes prevents the electrical breakdown. The accumulation of charge carriers on the dielectric surface produces an electric field with opposite direction to the applied field. These discharges in cellular polymers are responsible for charge accumulation on the cavities of the polymer and can be detected by its light emission [5]. They can also be considered as a particular case of the well-known partial discharges in high-voltage insulation [7].

After this pooling process, cellular polymers exhibit a high piezoelectric coefficient d_33_ [8,9]. Compared to ceramic piezoelectric devices, these polymers have high flexibility [10] and compared to piezoelectric polymers they show higher piezoelectric d_33_ constants, although less thermal stability. One difficulty of producing piezoelectric cellular polymer films is the charging method. Corona discharge has been one of the most traditional charging methods to provide piezoelectricity on cellular polymers [11,12]. The method had been optimized with by introducing the triode setup [13] which allows for a better control on the applied surface voltage. The implementation of corona discharge methodology has also been improved with a double charge exposition [14]. On the other hand, corona discharge has some disadvantages for industrial implementations due to the presence of high-voltage DC corona discharge, the dependence on environmental parameter and the difficulty to charge large areas.

Many works had tried to improve the charge accumulation inside the polymer structure, such as the inflation of the internal cavities by applying high-pressure treatment [15]. This process has led to two improvements: first, on the capability of the charge storage of the material and second, on their mechanical structure modifying its young modulus. Some improvements on polymer chain molecular structure were also made obtaining a glass transition temperature polymer increase [16]. Recent works have been focused on thermal behavior improvement [17] and material doping with mineral fillers [18].

In this work, a new contact electrode based on a series dielectric barrier for charging polypropylene foam films has been studied. This method allows for the use of a well-controlled geometry and can charge large surfaces easily. Section 2 presents the theoretical model. In Section 3 experimental, charging setup, sample conditioning, and piezoelectric coefficient measurement setup are shown. Measurements results of d_33_ coefficient mapping are discussed in Section 4 and are compared with the corona charging.

## 2. Theoretical Modelling

### 2.1. Samples with Metallized Electrodes vs. Corona Charging

In order to trigger the internal discharges in the voids, a high external electric field has to be applied to the samples. Generally, this is done by means of a corona discharge applied over the sample which is grounded on one side. Corona charging of samples induces a high surface charge density in the samples and thus a high internal electric field. The thin film has to be conductive to electrode free, at least on the surface in front of the corona discharge (the top surface). The grounded surface (bottom surface) can be metallized or not. One could think about metallizing both sides of the sample and directly applying a high voltage between the electrodes. However, this usually does not work properly and breakdown is reached before a high number of internal discharges are triggered. Breakdown usually happens in the center of the electrodes; thus, a border effect of the electrode is not predominant in this breakdown mechanism. Very low d_33_ constants can be obtained if the sample is not broken by electrical breakdown. In opposition to this, when charging with corona discharge, many internal discharges are triggered easily before breakdown happens in the sample.

**Figure 1:**
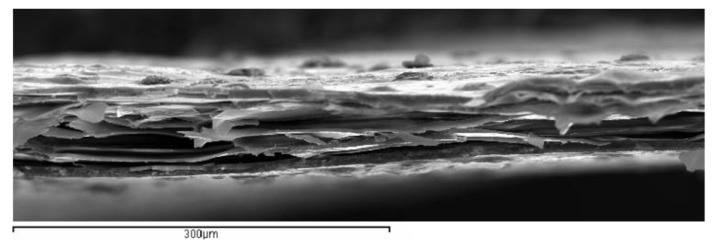
Image of a cross-section of the thin polypropylene films.

**Figure 2:**
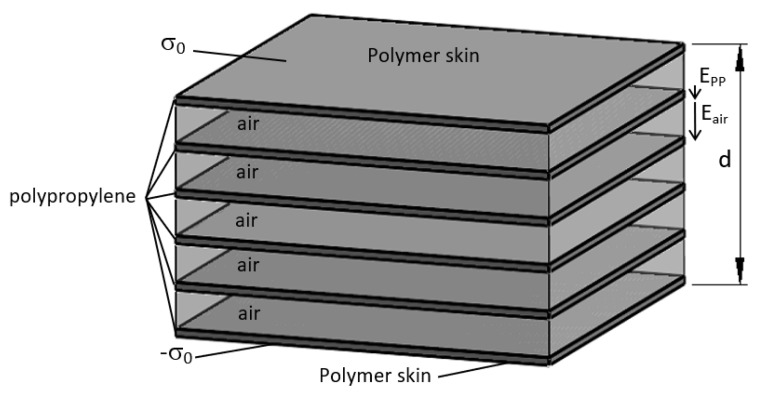
One-dimensional model of the sample charged with a surface charge $\sigma_{0}$.

To illustrate the situation, a one-dimensional model as proposed by Sessler [2] can help in understanding the different behavior in both cases. In our samples, there are an average of five cells in the thickness of the sample (Figure 1) which can be represented in the one-dimensional model (Figure 2).

If both sides of the electrode are metallized and an external electric voltage V is applied but no internal discharges happen, then the electric field in the polymer is:

(1) $$E_{\mathrm{pp}}=\frac{\sigma_{0}}{\varepsilon_{r}\varepsilon_{0}}$$

The electric field in the air in the voids is:

(2) $$E_{\mathrm{air}}=\frac{\sigma_{0}}{\varepsilon_{0}}$$

Thus, we have:

(3) $$V=\frac{\sigma_{0}}{\varepsilon_{r}\varepsilon_{0}}d\left(1-\alpha \right)+\frac{\sigma_{0}d\alpha }{\varepsilon_{0}}=\frac{\sigma_{0}}{\varepsilon_{r}\varepsilon_{0}}d\left(1-\alpha +{\alpha \varepsilon }_{r}\right)$$

where:

$\sigma_{0}$ is the charge supplied by the high-voltage generator.
$\varepsilon_{r}$ is the relative dielectric constant of the polymer.
$\varepsilon_{0}$ is the dielectric constant of vacuum.
α is the porosity of the sample defined as the ratio of the air volume in the sample divided by the total volume of the sample, typically 0.6.

If internal discharges happen in the sample, then an internal surface charge appears in the cells, thus reducing the electric field in the air. However, because of the high-voltage supply the applied voltage is constant and the initial surface charge $\sigma_{0}$ is increased to σ. In this new situation, it can be written as:

(4) $$V=\frac{\sigma }{\varepsilon_{r}\varepsilon_{0}}d\left(1-\alpha +{\alpha \varepsilon }_{r}\right)-\overset{m}{\underset{i}{\sum }}\frac{\sigma_{i}}{\varepsilon_{0}}\alpha \frac{d}{n}=\;\frac{\sigma }{\varepsilon_{r}\varepsilon_{0}}d\left[1-\alpha +{\alpha \varepsilon }_{r}-\overset{m}{\underset{i}{\sum }}\frac{\sigma_{i}{\alpha \varepsilon }_{r}}{\sigma n}\right]$$

where:

n is the total number of cells in the thickness of the sample (5 in Figure 2).
m is the total number of cells where an internal discharge happened (m ≤ n).
$\sigma_{i}$ is the surface charge created by internal discharges in the void.
σ is the new surface charge provided by the high-voltage supply.

Let us assume that $\sigma_{i}$ is the same in all the cells, then we have:

(5) $$V=\frac{\sigma }{\varepsilon_{r}\varepsilon_{0}}d\left[1-\alpha +{\alpha \varepsilon }_{r}-m\frac{\sigma_{i}{\alpha \varepsilon }_{r}}{\sigma n}\right]$$

As the voltage is the same in Equations (3) and (5), we can obtain the relation of the new surface charge σ to the first one $\sigma_{0}$:

(6) $$\frac{\sigma }{\sigma_{0}}=\frac{1-\alpha +{\alpha \varepsilon }_{r}\;}{1-\alpha +{\alpha \varepsilon }_{r}-m\frac{\sigma_{i}{\alpha \varepsilon }_{r}}{\sigma n}}$$

Or else:

(7) $$\sigma =\sigma_{0}\left[1+\frac{m\frac{\sigma_{i}{\alpha \varepsilon }_{r}}{\sigma n}}{1-\alpha +{\alpha \varepsilon }_{r}-m\frac{\sigma_{i}{\alpha \varepsilon }_{r}}{\sigma n}}\right]$$

To simplify the expression, let us assume that the electric field in the voids is completely cancelled, i.e., $\sigma_{i}=\sigma$. Then,

(8) $$\sigma =\sigma_{0}\left[1+\frac{m\frac{{\alpha \varepsilon }_{r}}{n}}{1-\alpha +{\alpha \varepsilon }_{r}-m\frac{{\alpha \varepsilon }_{r}}{n}}\right]$$

Under these assumptions, if an internal discharge has occurred in all the voids (m = n) we have:

(9) $$\sigma =\sigma_{0}\left[1+\frac{{\alpha \varepsilon }_{r}}{1-\alpha }\right]$$

That means, for a porosity of 0.6 and an $\varepsilon_{r}$ of 2.1, that the new electric field in the polypropylene, ${E^{\prime }}_{\mathrm{pp}}$, is increased by a factor of 4.15 compared to the field without internal discharges:

(10) $${E^{\prime }}_{\mathrm{pp}}=\frac{\sigma_{0}\left[1+\frac{{\alpha \varepsilon }_{r}}{1-\alpha }\right]}{\varepsilon_{r}\varepsilon_{0}}=E_{\mathrm{pp}}\left[1+\frac{{\alpha \varepsilon }_{r}}{1-\alpha }\right]=4.15{\;E}_{\mathrm{pp}}$$

It can be easily shown from (8) that if breakdown happens only in one cell out of 5, the theoretical factor is 1.18. If it happens in 2, 3, or 4 cells the factors are 1.44, 1.83, and 2.54 respectively.

According to the Paschen law [19], the voltage to produce a breakdown at 1 atm in an air gap of 12 μm (the thickness of one cell) is roughly 305 V, and an electric field of 25.4 MV/m. That means that the electric field in the polypropylene before breakdown in the cells according to (1) and (2) is 12.10 MV/m. With the assumption made in the Equation (10), after breakdown in all the cells, the electric field can theoretically reach a maximum of 50.21 MV/m which is above the breakdown strength of polypropylene (20 MV/m). This can explain why it is difficult to induce discharges in the internal cells without producing breakdown of the sample when both sides are metallized and the sample is charged at constant voltage.

**Figure 3:**
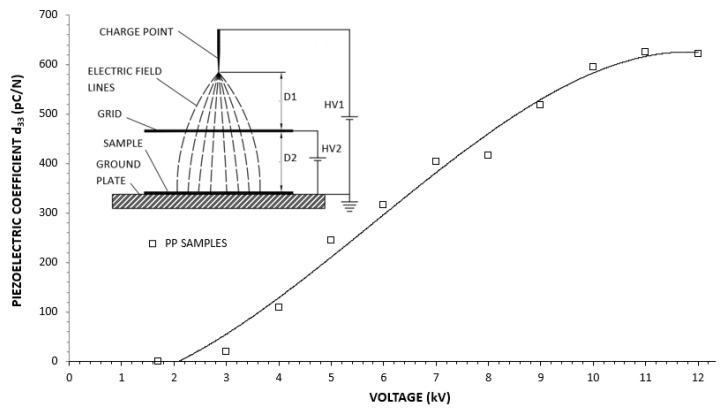
Piezoelectric coefficient d_33_ vs. grid potential in a triode corona charging setup.

The situation is different under corona charging. The surface voltage of the sample is not constant. In fact, after internal discharges, the electric potential of the surface is reduced (see e.g., [20]). During corona discharge, the surface charge is roughly constant, especially if it is charged with a triode system. Thus internal discharges do not increase the electric field in the polymer. This is why it is possible to trigger a large number of internal discharges without complete breakdown of the sample. Of course, breakdown can also happen if the surface potential of the sample is too high but it is possible to trigger many internal discharges in the voids before breakdown as shown by the d_33_ constants obtained. For example, when charging our samples with a triode corona arrangement, as shown in Figure 3, if we plot the d_33_ constant against the grid potential, it can be noticed that d_33_ constants are obtained from 3 kV to 12 kV of the grid potential. Over 13 kV no further improvement of the d_33_ constant is obtained, and breakdown of the sample happens at 16 kV. However, if the sample is metalized in both sides and connected to a high-voltage generator, breakdown happens at 2 kV approximatively and very low d_33_ constants (less than 10 pC/N) are obtained for lower voltages.

### 2.2. Dielectric Layer in Series with the Sample

**Figure 4:**
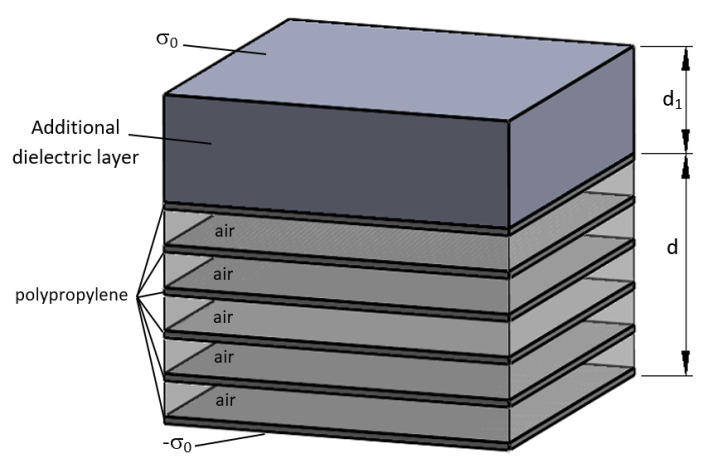
One-dimensional model of the sample in series with a dielectric layer charged with a surface charge $\sigma_{0}.$

In our proposal, a thick layer of a dielectric is inserted between the high-voltage electrode and the surface of the sample (Figure 4). Then, the applied voltage without internal discharges can be written as:

(11) $$V=\frac{\sigma_{0}}{\varepsilon_{r1}\varepsilon_{0}}d_{1}+\frac{\sigma_{0}}{\varepsilon_{r}\varepsilon_{0}}d\left(1-\alpha +{\alpha \varepsilon }_{r}\right)$$

where

$d_{1}$ is the thickness of the new layer.
$\varepsilon_{r1}$ is the relative dielectric constant of the additional dielectric layer.

Let us consider that the thickness $d_{1}=\mathrm{kd}$

Under the same assumptions as in (9), and, for the sake of simplicity, let us consider $\varepsilon_{r1}=\varepsilon_{r}$. We have:

(12) $$\sigma =\sigma_{0}\left[1+\frac{{\alpha \varepsilon }_{r}}{k+1-\alpha }\right]$$

For a factor k = 4 as in one of our electrodes, the electric field is only increased by a factor of 1.28 in the worst case which is considerably less. Of course, the applied voltage V has to be higher to reach breakdown in the cells, but the electric field should theoretically be the same in the air and in the polymer as in the case without the dielectric layer. The advantage of this system is that the effect of air breakdown in the cells does not increase the electric field significantly in the polymer. Thus, again, it is possible to apply high electric fields to the sample without breakdown and to obtain similar results in the case of corona charging, as it is shown below.

## 3. Materials and Methods

### 3.1. Sample Conditioning

The foamed polypropylene film used in this work is produced by the company Sekisui Alveo B.V. (Roermond, the Netherlands). The film was thermally modified by biaxial stretching, reducing its thickness by 90% from 1 mm to 100 μm [21]. This biaxial stretching increases the piezoelectric properties of the material compared to the non-stretched samples. Original samples have a relation between air and polymer with about 80% of air and 20% of polypropylene and Young’s modulus between 2 and 3 MPa. After biaxial stretching, samples have a porosity of around 60%. As a consequence the samples increase their density up to 400 kg/m^3^ and Young’s modulus was also reduced to 0.2 to 0.3 MPa for a thickness of 100 µm or less.

It can be observed how the original nearly spherical shape of air voids is modified into flat ones (Figure 1) allowing an increase of charge accumulation on it. After biaxial thermal stretching, samples were cut in squares of about 70 mm × 70 mm divided in a grid of 25 areas (Figure 7). These areas are used to measure the piezoelectric d_33_ constant to perform a mapping of the surface. All the samples were metallized by sputtering with silver on the grounded side before dielectric barrier charging. After charging, the sample was again metallized twice to get a good electrical conductivity on the top electrode. Then the d_33_ constant was measured. The same result can be obtained if the sample is charged and then metallized on both sides.

### 3.2. Series Dielectric Layer Electrode

**Figure 5:**
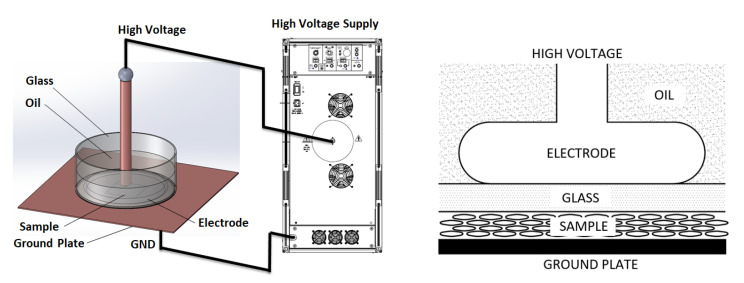
Experimental setup with a dielectric layer of glass and the high-voltage electrode surrounded by oil to avoid surface discharges.

In our previous measurements with corona charging [21], we obtained the maximum piezoelectric constant, which means the maximum number and strength of dielectric breakdowns in the internal voids of the polymer, in the range of 12 kV of the applied surface potential on the sample (Figure 3). When a dielectric layer is placed in series between the sample and a high-voltage electrode, this potential has to also reach the surface of the sample to obtain the same internal condition in the sample. This requires a high-electric potential on top of the dielectric layer, thus forming corona discharges on the high-voltage electrode. The first dielectric layer that was used was a 1.95-mm thick glass (Figure 5). To avoid surface discharges on the glass, the high-voltage electrode was immersed in dielectric oil (Tensión Centauro, Repsol, Madrid, Spain).

A high-voltage amplifier Trek model 50/12 (Advanced Energy AE, Littlehampton, West Sussex, UK) was the DC voltage supply. The high-voltage electrode was designed to minimize the partial discharges on air. The sample is located between the glass and the ground plate. The high-voltage electrode has a diameter of 40 mm and the sample is a 70-mm square. The glass has a diameter of 150 mm and completely covers the sample. Measurements were carried out at room temperature. First positive results were obtained with this setup.

**Figure 6:**
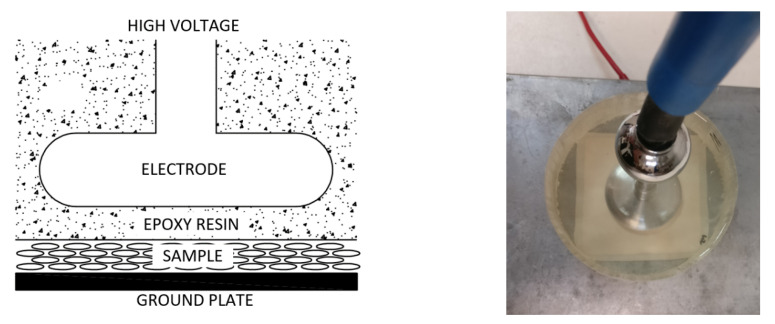
Experimental setup with a dielectric layer of epoxy resin and the electrode surrounded by epoxy resin too.

Then, a second setup was built to improve the dielectric strength of the system. The oil and glass were replaced by epoxy resin (EC-141 Epoxy resin, Glaspol Composites S.L. Valencia, Spain) (Figure 6). This material has an electrical permittivity of 2.5 and a high dielectric strength of 35 kV/mm. Two thicknesses of the epoxy layer between the electrode and the sample were used: 400 μm and 300 μm.

Again, the high-voltage electrode has a diameter of 40 mm and the sample is 70 mm square with a metallized ground electrode of 61 mm square. The diameter of the resin is 90 mm and overlaps nearly the entire surface of the sample. It covers completely the area over the metallized ground electrode. In Figure 6, a top view of the setup with the epoxy resin electrode, the high-voltage electrode, and the sample is shown.

### 3.3. Piezoelectric Constant Measurement

The quasi-static piezoelectric d_33_ coefficient [22] was measured by a customized design setup [21]. On this setup an actuator based on a solenoid pressed the sample by a controlled force system generator. A signal function generator (Tektronics model AFG 3251, Beaverton, OR, USA) coupled to an electronic current amplifier circuit is responsible for the magnitude and pattern of the current in the solenoid and thus the magnitude and pattern of the force on the sample. To allow a mechanical recovery of the sample between pulses, the time interval between mechanical pulses was 1.5 s. This time is directly in relation with the elastic behavior of the polypropylene foam. Dynamic test can be performed by changing the parameters like frequency and force. The current in the sample is first integrated by an analog electronic integration circuit based on an operational amplifier OPA129 and then measured by a Yokogawa DL9040 oscilloscope (Tokyo, Japan). The system was calibrated with a commercial coulombmeter Keithley 6514 (Cleveland, OH, USA).

The applied force on the samples was 3 N. This value was selected because it is the optimal for the maximum d_33_ coefficient response of these samples [21]. The force was applied with a plunger and measured with a load cell in series with the plunger.

**Figure 7:**
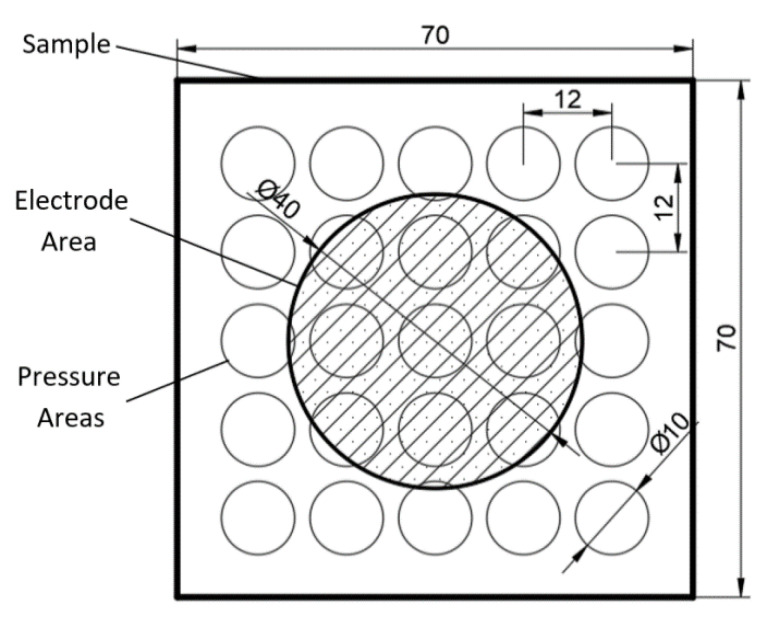
Sample measurement points distribution, and charging electrode over sample (dimensions in mm).

An image of the sample, the electrode and the pressure areas from a top view is shown in Figure 7. The pressure areas have a surface of 80 mm^2^. These pressure areas allow for a more representative measurement because it involves several cells under the plunger, decreasing the influence of the heterogeneity structure of the material. A mapping of the surface distribution of the d_33_ constant can be obtained by manually moving the plunger across the surface of the sample in 25 positions as shown in Figure 7 by circles.

## 4. Results and Discussion

### 4.1. Glass Layer Charging

**Figure 8:**
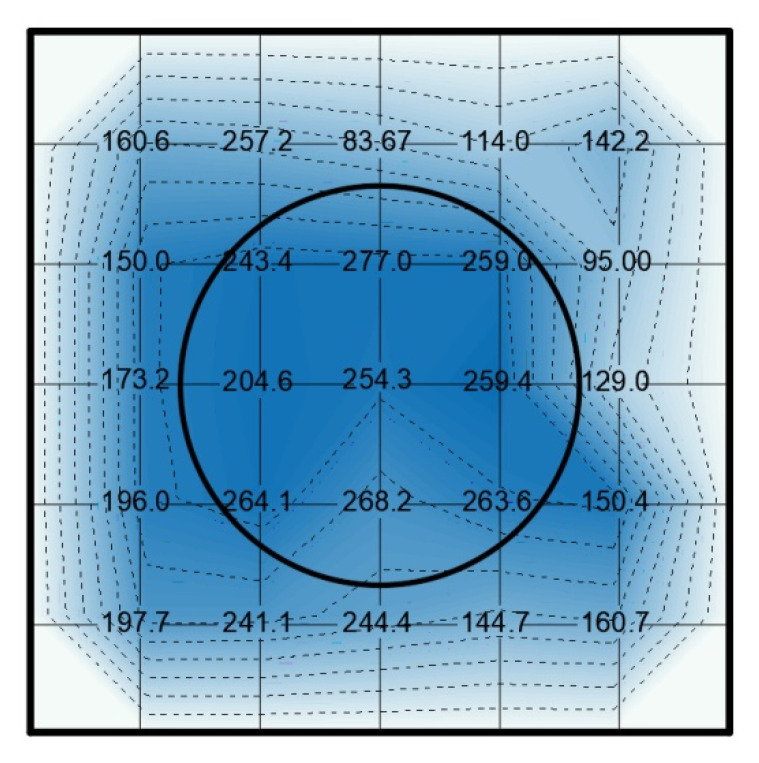
Example of spatial distribution of d_33_ constant in a sample charged with a glass dielectric layer with a maximum of 277 pC/N. The numbers indicate the value of the d_33_ constant in the measurement points and the circle the position of the high-voltage electrode.

Voltages of up to 50 kV for 30 s were required to obtain maximum piezoelectric d_33_ coefficients of about 200 pC/N and a maximum of 254 pC/N. Lower voltages did not provide high piezoelectric constants. Glass breakdown happened in some cases with 50 kV. An example of spatial distribution of the d_33_ constant can be found in Figure 8. The distribution of the d_33_ constant is coherent with the shape of the electrode and the electric field distribution in the sample. A maximum is obtained close to the border of the electrode probably due to an enhancement effect and the anisotropy of the sample. This setup exhibited a large dispersion in results and breakdown of the glass was reached in some tests.

In Figure 8, d_33_ coefficient measurements can be seen on a sample activated by dielectric barrier with glass layer. The distribution of the piezoelectric constant is shown by means of a graphical representation by colors and d_33_ isolines interpolated between the measuring points and considering a zero value in the borders. The high-voltage electrode position is represented on the center of the sample where the highest values of d_33_ are expected. However, the influence of the electrode extends outside the electrode border. The rounded edge of the electrode, in this case with a radius of 5 mm, can explain this behavior.

The distribution of the d_33_ coefficient is not only determined by the internal electric field distribution but also by the homogeneity of the foam structure of the film, that is the density of the bubbles in specific areas and the thickness variations of the sample. Thus a certain degree of dispersion of the d_33_ coefficient was found in all samples.

### 4.2. Epoxy Resin Electrode Charging

**Figure 9:**
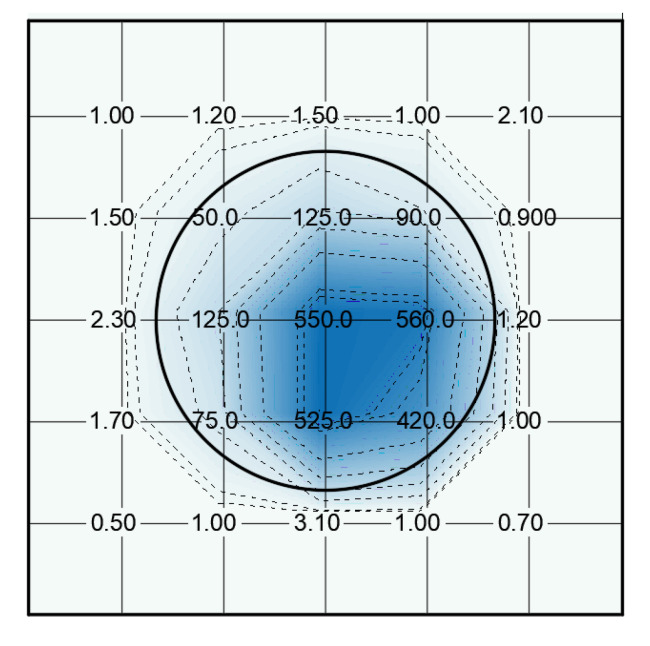
Example of spatial distribution of d_33_ constant in a sample charged with an epoxy dielectric layer with an epoxy thickness of 400 µm. The numbers indicate the value of the d_33_ constant in the measurement points and the circle the position of the high-voltage electrode.

**Figure 10:**
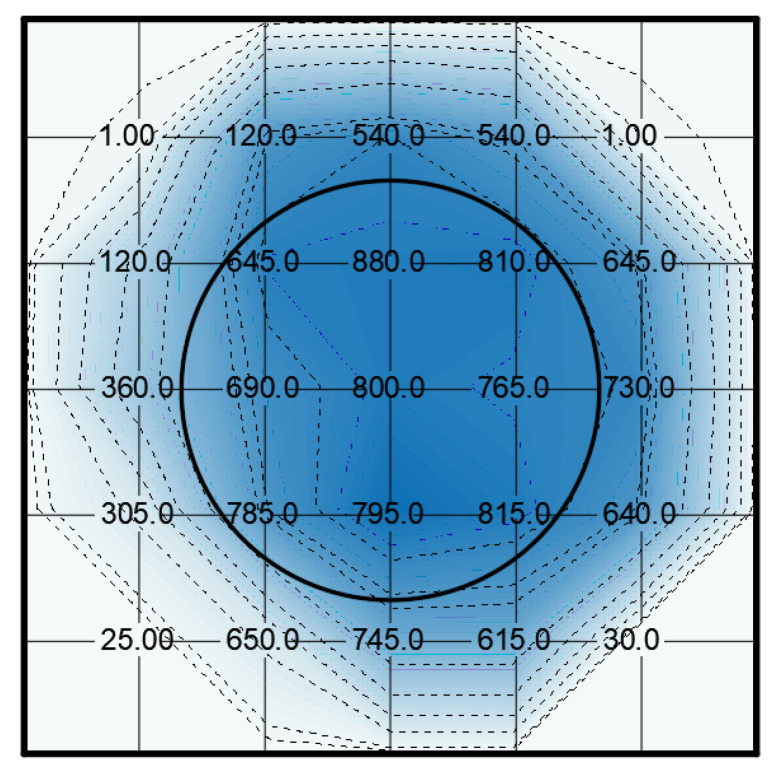
Example of spatial distribution of d_33_ constant in a sample charged with an epoxy dielectric layer with an epoxy thickness of 300 µm. The numbers indicate the value of the d_33_ constant in the measurement points and the circle the position of the high-voltage electrode.

Maximum piezoelectric d_33_ coefficient of 560 pC/N were found on samples that were activated by the resin electrode of 400 μm thickness whereas the d_33_ constant reached maximum values of 880 pC/N with the resin electrode of 300 μm thickness. Both thicknesses allow for the DC voltage to be reduced to 30 kV for 30 s. A higher voltage can produce breakdown of the electrode. As can be seen in Figure 9 and Figure 10, the distribution of the d_33_ constant is related to the electrode position as in the previous case with, again, a maximum close to the border of the electrode. The measurements show an increment on the d_33_ constants due to the reduction of the thickness of the resin electrode. Again, the cellular structure of the film has some randomness that affects the charge distribution.

The effect of environmental humidity on the results obtained should be noted because of the water absorption by the epoxy resin which modifies its electrical properties. A higher conductivity or polarization of the epoxy resin distorts the applied electric field and leads to lower and irregular results. The electrode had to be dried and kept under low controlled humidity before use. On the other hand, this electrode is preferred to the glass electrode because of its ease of handling for a smaller thickness, its charging stability, and its performance for future applications.

### 4.3. Estimated Surface Potential and Equivalence with Corona Charging

Results are consistent with the hypothesis that the series dielectric allows equivalent surface potentials to be applied to the samples as with a corona charging system.

By using Equation (11), it can be shown that, when applying a voltage V to the system formed by the dielectric in series with the sample, the theoretical electric field in the air of the internal voids is:

(13) $$E_{\mathrm{air}}=\frac{V}{\left[\frac{d_{1}}{\varepsilon_{r1}}+\frac{d\left(1-\alpha \right)}{\varepsilon_{r}}+\alpha d\right]}$$

Thus, the surface potential, $V_{\mathrm{Surf}}$, at the sample is:

(14) $$V_{\mathrm{surf}}=E_{\mathrm{air}}\alpha d+\frac{E_{\mathrm{air}}}{\varepsilon_{r}}\left(1-\alpha \right)d$$

It can be easily found with the same variables as previously:

(15) $$V_{\mathrm{surf}}=V\left[\frac{\left(1-\alpha +{\alpha \varepsilon }_{r}\right)\varepsilon_{r1}}{{k\varepsilon }_{r}+\left(1-\alpha +{\alpha \varepsilon }_{r}\right)\varepsilon_{r1}}\right]$$

Considering the three analyzed cases with the common parameters: $\alpha =0.6,\;d=100\;\mu m,{\;\varepsilon }_{r}=2.1$, we have the estimated theoretical applied surface potential with the parameters and results shown in Table 1. When compared to Figure 3, the expected d_33_ constants with corona charging are approximately 450 pC/N, 580 pC/N, and 650 pC/N for 7.8 kV, 9.9 kV, and 12 kV, respectively. These values are consistent with those obtained by charging with the series dielectric layer.

Finally, a comparison between the presented method and corona charging method concerning the double charge method [14] was carried out. The method consists of applying the corona charging treatment on one side of the sample (without sputtering). Then the side exposed to corona discharge is metallized. The sample is turned around and charged again. Finally, the other side is metallized. This procedure gave good results in samples charged by corona discharge. These results were replicated by [16] calling it reverse charging method. They obtained an improvement of the piezoelectric constant of their materials.

The double charging was carried out with the dielectric series layer presented here but no difference on piezoelectric d_33_ constant between the two methods was observed. This may indicate that the results obtained with the double charging method or reverse charging method are dependent on the external corona discharge physics and not on the sample internal discharges.

## 5. Conclusions

The charging of piezoelectric cellular polypropylene by dielectric barrier series layer is described. Results, electrode prototype, and theoretical analysis of the values are presented. The d_33_ piezoelectric constant of the samples was measured using our own design setup with spatial resolution in order to obtain a mapping of the d_33_ constant on the samples. Based on our previous works and experience with corona discharge in piezoelectric polymers, this charging method provides similar and better results. In addition, the suggested method has some advantages for future industrial applications because of its simple assembly and from an electrical point of view the usage of insulated DC high voltage. Two possible setups for charging the samples were tested based on glass and resin electrode respectively, resulting in the last one showing a better behavior reaching values of up to 880 pC/N. Optimization of the epoxy thickness layer provided better results. Good homogeneity of the surface distribution of the d_33_ constant on the area under the electrode was obtained. Based on this work, new electrodes with a dielectric series layer can be developed to improve the results by using dielectrics with a high dielectric constant, a high dielectric strength, and/or a lower thickness.

## Figures and Tables

**Table 1:** Maximum d_33_ constant obtained and estimated theoretical applied surface potential of the sample with the three different electrodes.

	Parameters	d_33_ Constant	Theoretical Applied Surface Potential
Glass	$d_{1}=1.95\;\mathrm{mm}$ (k = 19.5) $\varepsilon_{r1}=4.8$, V = 50 kV	254 pc/N	7.8 kV
Epoxy	$d_{1}=400\;\mu m$ (k = 4) $\varepsilon_{r1}=2.5$, V = 30 kV	650 pc/N	9.9 kV
Epoxy	$d_{1}=300\;\mu m$ (k = 3) $\varepsilon_{r1}=2.5$, V = 30 kV	880 pc/N	12 kV

## Data Availability

The data presented in this study are available on request from the corresponding author.
